# Predictors associated with unplanned hospital readmission of medical and surgical intensive care unit survivors within 30 days of discharge

**DOI:** 10.1186/s40560-018-0284-x

**Published:** 2018-03-01

**Authors:** Tetsu Ohnuma, Daisuke Shinjo, Alan M. Brookhart, Kiyohide Fushimi

**Affiliations:** 1 Department of Health Policy and Informatics, Tokyo Medical and Dental University Graduate School, 1-5-45 Yushima, Bunkyo-ku, Tokyo, 1138519 Japan; 2 Department of Epidemiology, Gillings School of Global Public Health, University of North Carolina, Chapel Hill, USA; 3 The Database Center of the National University Hospital, The University of Tokyo Hospital, Tokyo, Japan

**Keywords:** Hospital readmission, Rehospitalizations, Intensive care unit, Critical illness, Predictors, Outcomes research

## Abstract

**Background:**

Reducing the 30-day unplanned hospital readmission rate is a goal for physicians and policymakers in order to improve quality of care. However, data on the readmission rate of critically ill patients in Japan and knowledge of the predictors associated with readmission are lacking. We investigated predictors associated with 30-day rehospitalization for medical and surgical adult patients separately.

**Methods:**

Patient data from 502 acute care hospitals with intensive care unit (ICU) facilities in Japan were retrospectively extracted from the Japanese Diagnosis Procedure Combination (DPC) database between April 2012 and February 2014. Factors associated with unplanned hospital readmission within 30 days of hospital discharge among medical and surgical ICU survivors were identified using multivariable logistic regression analysis.

**Results:**

Of 486,651 ICU survivors, we identified 5583 unplanned hospital readmissions within 30 days of discharge following 147,423 medical hospitalizations (3.8% readmitted) and 11,142 unplanned readmissions after 339,228 surgical hospitalizations (3.3% readmitted). The majority of unplanned hospital readmissions, 60.9% of medical and 63.1% of surgical case readmissions, occurred within 15 days of discharge. For both medical and surgical patients, the Charlson comorbidity index score; category of primary diagnosis during the index admission (respiratory, gastrointestinal, and metabolic and renal); hospital length of stay; discharge to skilled nursing facilities; and having received a packed red blood cell transfusion, low-dose steroids, or renal replacement therapy were significantly associated with higher unplanned hospital readmission rates.

**Conclusions:**

From patient data extracted from a large Japanese national database, the 30-day unplanned hospital readmission rate after ICU stay was 3.4%. Further studies are required to improve readmission prediction models and to develop targeted interventions for high-risk patients.

**Electronic supplementary material:**

The online version of this article (10.1186/s40560-018-0284-x) contains supplementary material, which is available to authorized users.

## Background

Hospital readmission adversely affects patients and healthcare systems. The rate of hospital readmission is an important problem faced by the Japanese health care system. Readmission may occur because of unresolved acute illness, ongoing chronic illness, the development of new medical complications, or from gaps in outpatient care [1, 2]. In the post-intensive care setting, early hospital readmission is an indicator of poor-quality care of these vulnerable patients. Indeed, patients admitted to intensive care units (ICUs) who survive to hospital discharge have a higher 6-month mortality rate post-discharge than patients hospitalized without critical illness. In addition, they experience significant morbidity subsequent to their ICU stay [3, 4].

Unplanned hospital readmission is a more informative marker of clinical deterioration than all-cause hospital readmission since planned readmissions do not indicate poor-quality care [5, 6]. Only one study assessed predictors associated with readmission of critically ill patients using claims data [2]. In that study, admissions scheduled at least 24 h in advance were considered operationally planned admissions and were not included in the definition of re-hospitalization; hence, this definition might have underestimated the true prevalence of readmission.

Identifying predictors associated with unplanned hospital readmission may inform policymakers and facilitate identification of high-risk patients to be targeted for future interventions. However, such data are currently lacking in Japan. Therefore, the aim of this study was to determine the predictors associated with unplanned hospital readmission of ICU survivors within 30 days of hospital discharge by using routine admissions data from the Japanese Diagnosis Procedure Combination (DPC) database. In addition, because we hypothesized that medical and surgical patients would have different predictors for readmission, we analyzed medical and surgical patients separately.

## Methods

### Data source

The Tokyo Medical and Dental University ethics committee approved this study and waived the requirement for informed consent. The DPC database is a Japanese case-mix classification system linked to a payment system. Details of the DPC have been described elsewhere [7, 8]. In short, by 2011, data from more than 1400 acute care hospitals were included in the DPC database, covering approximately 50% of patients discharged from all Japanese hospitals. The database includes data on baseline patient information; diagnosis, according to the International Classification of Diseases and Injuries, 10th revision (ICD-10); medical procedures; medications; materials; discharge destination; and information regarding hospital readmission.

### Study population

Data of all patients admitted to ICU between April 2012 and February 2014 who survived to hospital discharge were retrospectively extracted from the DPC database using ICU bed utilization billing codes. Patients who required only step-down unit care (high care unit care) were not included. Exclusion criteria were as follows: missing data on admission, admission dates, or discharge dates; age less than 18 years; and hospital length of stay (LOS) > 365 days. Data of patients who were readmitted to other hospitals were unavailable because patient registration numbers differ at each hospital.

### Data collection and classification

We divided the hospital readmission cohort into medical and surgical cases. Patients who underwent any surgery were considered surgical cases. All other patients were considered medical cases. The cause, type (planned or unplanned), and outcome of hospital readmission were obtained from the DPC database. Planned hospital readmissions were not counted as re-hospitalization events in this analysis because we were interested in investigating hospital readmissions that are potentially preventable. Subsequent hospital readmissions were counted if they met the inclusion criteria.

We used the Charlson comorbidity index (CCI) to quantify the burden of comorbid illness [9]. Level of consciousness was assessed at admission using the Japan Coma Scale score: 0 (alert), 1–3 (delirious), 10–30 (somnolent), and 100–300 (comatose) [10]. The category of primary diagnosis of each patient was defined using ICD-10 coding, as presented in Additional file 1: Table S1. The categories considered were cardiac, respiratory, neurologic, gastrointestinal, malignancy, metabolic and renal, and other. Hospital-level characteristics, academic status (teaching or non-teaching), and size (< 399, 400–799, and > 800 beds) were captured. Hospital LOS was categorized into four groups: 1–15, 16–30, 31–45, and > 46 days.

Data on the use of a vasopressor, stress ulcer prophylaxis, blood products (packed red blood cells [pRBC], fresh frozen plasma [FFP], and platelets), low-dose steroids, total parental nutrition (TPN), any antibiotic, renal replacement therapy (RRT), and ventilation were extracted from the DPC database.

### Outcomes

The primary outcome was unplanned hospital readmission within 30 days of discharge following the index hospitalization. Unplanned readmission rates for the medical and surgical cohorts were calculated by dividing the number of survivors readmitted by the total number of survivors in each cohort. Diagnosis on hospital readmission was categorized using primary ICD-10 diagnosis categories.

### Statistical analysis

Data were presented as means ± standard deviation, medians and interquartile ranges (IQR), or percentages, as appropriate. Length of ICU stay was calculated based on the number of days billed. We used multivariable logistic regression to identify predictors associated with 30-day unplanned hospital readmission. The results were presented as odds ratios and 95% confidence intervals. We examined the following co-variables: teaching hospital; hospital size; age; sex; CCI score; category of primary diagnosis; coma on admission; hospital LOS; discharge destination; and requirement for vasopressors, stress ulcer prophylaxis, blood product (pRBC, FFP, platelet) transfusion, steroids, anticoagulant therapy, TPN, antibiotics, RRT, ventilation, and tracheostomy. Multicollinearity between covariates was assessed using variance inflation factor and tolerance values. To assess the model performance, we calculated the area under the receiver operating characteristic curve. The curves measured the model’s ability to distinguish between readmission and no readmission by evaluating the C-statistic. A C-statistic equals to one indicates perfect discrimination, whereas a C-statistic of 0.5 indicate that the ability of the model to discriminate is due to chance. All analyses were performed using R statistical software, version 3.3.0 (R Foundation for Statistical Computing, Vienna, Austria).

## Results

### Characteristics of survivors

**Fig. 1:**
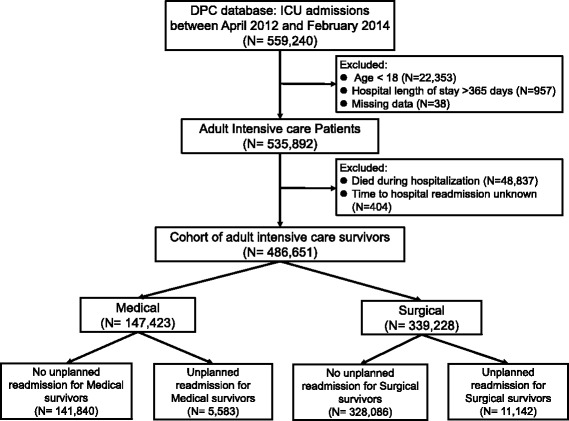
Selection of adult survivors of medical and surgical intensive care unit admission

We enrolled 559,240 patients from 502 hospitals who were admitted to ICU for at least 1 day between April 2012 and February 2014. Overall hospital mortality was 9.1% (medical, 18.1% and surgical, 6.5%). After excluding patients for the reasons outlined in Fig. 1, the final cohort included 486,651 ICU patients who survived to hospital discharge. The 30-day unplanned hospital readmission rates following medical and surgical admissions were 3.8% (5583 of 147,423) and 3.3% (11,142 of 339,228), respectively. In the medical group, 5102 (3.6%) of all unique patients admitted were readmitted once, 383 (0.3%) were readmitted twice, and 77 (0.1%) were readmitted three times or more. In the surgical group, 10,573 (3.2%) of all unique patients admitted were readmitted once, 520 (0.2%) were readmitted twice, 45 (0.01%) were readmitted three times or more.

The characteristics of the cohorts of medical and surgical survivors are shown in Tables 1 and 2. The mean age of patients was 69.7 ± 14.4 years for the medical cohort and 68.2 ± 14.3 years for the surgical cohort. The median ICU stay was 2 (IQR, 1–4) days for medical and 1 (IQR, 1–3) day for surgical cases, and the median length of hospital stay was 17 (IQR, 10–32) days for medical and 22 (IQR, 15–37) days for surgical cases. Medical patients were discharged home (76.3%), transferred to another hospital (20.6%), or discharged to a skilled nursing facility (2.2%). On the other hand, 84.5% of surgical patients were discharged home, 13.9% were transferred to another hospital, and 0.8% were discharged to a skilled nursing facility.

**Table 1:** Baseline characteristics of survivors in medical and surgical intensive care units

Characteristic	Medical		Surgical	
	No unplanned readmission	Unplanned readmission	No unplanned readmission	Unplanned readmission
	(*n* = 141,840)	(*n* = 5583)	(*n* = 328,086)	(*n* = 11,142)
Hospital characteristics				
Teaching, *n* (%)	23,329 (17.2)	794 (14.9)	100,516 (31.9)	2840 (26.6)
Size, *n* (%)				
< 399 beds	49,826 (36.7)	2012 (37.7)	74,587 (23.7)	2654 (24.9)
400–799 beds	62,135 (45.8)	2568 (48.1)	154,905 (49.2)	5575 (52.2)
> 800 beds	23,715 (17.5)	759 (14.2)	85,576 (27.2)	2451 (22.9)
Age, mean (SD), year	69.7 (14.4)	70.1 (14.2)	68.2 (14.3)	68.7 (14.2)
Men, *n* (%)	89,565 (63.1)	3450 (61.8)	193,766 (59.1)	7109 (63.8)
BMI, mean (SD)	22.9 (4.4)	22.2 (4.2)	22.7 (4.3)	22.4 (4.0)
CCI, median (IQR)	1 (0–2)	1 (0–2)	1 (0–2)	1 (0–2)
0, *n* (%)	52,063 (36.7)	1686 (30.2)	137,257 (41.8)	3836 (34.4)
1, *n* (%)	45,830 (32.3)	1683 (30.1)	87,625 (26.7)	3040 (27.3)
2, *n* (%)	26,809 (18.9)	1251 (22.4)	56,597 (17.3)	2146 (19.3)
≥ 3, *n* (%)	17,138 (12.1)	963 (17.2)	46,607 (14.2)	2120 (19.0)
Primary category of diagnosis on admission *n* (%)				
Cardiac	69,882 (49.3)	2792 (50.0)	49,981 (15.2)	1807 (16.2)
Respiratory	8626 (6.1)	549 (9.8)	5853 (1.8)	257 (2.3)
Neurologic	31,903 (22.5)	799 (14.3)	61,290 (18.7)	1822 (16.4)
Gastrointestinal	4336 (3.1)	252 (4.5)	25,473 (7.8)	1180 (10.6)
Malignancy	7158 (5.0)	330 (5.9)	138,309 (42.2)	4645 (41.7)
Metabolic and renal	5055 (3.6)	297 (5.3)	6507 (2.0)	329 (3.0)
Other	14,880 (10.5)	564 (10.1)	40,673 (12.4)	1102 (9.9)
Coma on admission, *n* (%)	8697 (6.1)	285 (5.1)	6157 (1.9)	198 (1.8)
ICU stay, median (IQR), days	2 (1–4)	2 (1–5)	1 (1–3)	1 (1–3)
Hospital stay, median (IQR), days	18 (10–32)	21 (12–37)	22 (15–37)	26 (17–44)
1–15 days, *n* (%)	62,371 (44.0)	1936 (34.7)	93,430 (28.5)	2364 (21.2)
16–30 days, *n* (%)	41,160 (29.0)	1823 (32.7)	125,972 (38.4)	4125 (37.0)
31–45 days, *n* (%)	16,266 (11.5)	783 (14.0)	49,191 (15.0)	1994 (17.9)
> 45 days, *n* (%)	22,043 (15.5)	1041 (18.6)	59,493 (18.1)	2659 (23.9)
Discharge destination				
Home, *n* (%)	107,590 (75.9)	4572 (81.9)	276,386 (84.2)	9821 (88.1)
Other hospital, *n* (%)	29,937 (21.1)	698 (12.5)	46,441 (14.2)	1072 (9.6)
Skilled nursing facility, *n* (%)	3099 (2.2)	230 (4.1)	2458 (0.7)	154 (1.4)
Other, *n* (%)	1214 (0.9)	83 (1.5)	2800 (0.9)	95 (0.9)

*SD* standard deviation, *BMI* body mass index, *CCI* Charlson comorbidity index, *IQR* interquartile range, *ICU* intensive care unit

**Table 2:** Treatments received in medical and surgical intensive care units during hospitalization

Characteristic	Medical		Surgical	
	No unplanned readmission	Unplanned readmission	No unplanned readmission	Unplanned readmission
	(*n* = 141,840)	(*n* = 5583)	(*n* = 328,086)	(*n* = 11,142)
Vasopressor, *n* (%)	26,513 (18.7)	1136 (20.3)	93,209 (28.4)	3319 (29.8)
Stress ulcer prophylaxis, *n* (%)	107,214 (75.6)	4266 (76.4)	256,934 (78.3)	9277 (83.3)
Blood product usage				
pRBC, *n* (%)	15,930 (11.2)	891 (16.0)	109,216 (33.3)	4937 (44.3)
FFP, *n* (%)	2563 (1.8)	147 (2.6)	23,040 (7.0)	1006 (9.0)
Platelets, *n* (%)	4050 (2.9)	224 (4.0)	33,954 (10.3)	1468 (13.2)
Steroid use, *n* (%)	5812 (4.1)	303 (5.4)	20,556 (6.3)	838 (7.5)
Anticoagulant therapy, *n* (%)	86,280 (60.8)	3296 (59.0)	232,864 (71.0)	8163 (73.3)
TPN, *n* (%)	36,037 (25.4)	1574 (28.2)	130,747 (39.9)	4998 (44.9)
Antibiotic usage, *n* (%)	64,532 (45.5)	2972 (53.2)	269,500 (82.1)	9399 (84.4)
RRT				
Number, *n* (%)	9722 (6.9)	609 (10.9)	19,073 (5.8)	998 (9.0)
Duration, days, median (IQR)	3 (2–6)	3 (2–5)	4 (2–6)	3 (2–6)
Ventilation				
Number, *n* (%)	27,731 (19.6)	1459 (26.1)	82,005 (25.0)	3395 (30.5)
Duration, days, median (IQR)	4 (2–9)	4 (2–8)	2 (1–6)	2 (1–6)
Tracheostomy *n* (%)	2626 (1.9)	91 (1.6)	8978 (2.7)	295 (2.6)

*pRBC* packed red blood cells, *FFP* fresh frozen plasma, *TPN* total parental nutrition, *RRT* renal replacement therapy, *IQR* interquartile range

During the index hospitalization, the proportions of medical and surgical cases, respectively, requiring various treatments were as follows: vasopressors, 27 vs 43%; stress ulcer prophylaxis, 76 vs 78%; pRBCs, 11 vs 33%; low-dose steroids, 4 vs 7%. Ventilation was required for a median of 4 (2–9) days for medical and 2 (1–6) days for surgical patients, and 7% of medical and 6% of surgical patients received RRT.

### Timing of and reasons for unplanned hospital readmission

The majority of 30-day unplanned hospital readmissions occurred within 15 days of initial hospital discharge; 60.9% of the medical and 63.1% of the surgical readmissions occurred within 15 days, while 32% of readmissions in each cohort occurred within the first 7 days of discharge (Additional file 2: Figure S1). The most common categories of diagnosis assigned to ICU survivors at the time of unplanned hospital readmission were cardiac (31.5%) for medical cases and malignancy (19.6%) for surgical cases (Table 3). The in-hospital mortality rate was 10.5% for medical and 7.5% for surgical ICU survivors who were rehospitalized.

**Table 3:** Reasons for and outcomes of 30-day unplanned hospital readmissions of ICU survivors

Category of primary diagnosis, *n* (%)	Medical		Surgical	
	At readmission	At initial index admission	At admission	At initial index admission
	(*n* = 5583)	(*n* = 147,423)	(*n* = 11,142)	(*n* = 339,228)
Cardiac	1758 (31.5)	72,674 (49.3)	1298 (11.6)	51,788 (15.3)
Malignancy	345 (6.2)	7488 (5.1)	2184 (19.6)	14,2954 (42.1)
Respiratory	738 (13.2)	9175 (6.2)	1138 (10.2)	6110 (1.8)
Neurologic	636 (11.4)	32,702 (22.2)	1022 (9.2)	63,112 (18.6)
Gastrointestinal	640 (11.5)	4588 (3.1)	1298 (11.6)	26,653 (7.9)
Metabolic and renal	575 (10.3)	5352 (3.6)	991 (8.9)	6836 (2.0)
Other	891 (16.0)	15,444 (10.5)	2457 (22.1)	41,775 (12.3)
Outcomes				
Hospital length of stay, median (IQR)	15 (8–28)		14 (8–26)	
Hospital mortality, %	585 (10.5)		836 (7.5)	

*ICU* intensive care unit, *IQR* interquartile range

### Predictors associated with 30-day unplanned hospital readmission

In the multivariable logistic regression analysis, the factors associated with increased 30-day unplanned hospital readmission rate were similar for medical and surgical patients (Table 4 and Additional file 1: Table S2). The area under the receiver operating characteristic curve was 0.64 (0.63–0.65) for the medical patients and 0.62 (0.61–0.63) for the surgical patients (Additional file 3: Figure S2).

**Table 4:** Predictors associated with 30-day unplanned hospital readmission of ICU survivors

	Medical	Surgical
	(*n* = 147,840)	(*n* = 339,228)
Variable	OR (95% CI)	OR (95% CI)
Teaching hospital	–	0.79 (0.75–0.84)
Hospital size		
< 399 beds	Reference	Reference
400–799 beds	0.98 (0.93–1.05)	0.99 (0.94–1.04)
> 800 beds	0.77 (0.69–0.87)	0.90 (0.84–0.97)
CCI	1.10 (1.08–1.12)	1.05 (1.04–1.06)
Category of primary admission diagnosis		
Cardiac	Reference	Reference
Respiratory	1.30 (1.17–1.45)	1.51 (1.31–1.75)
Neurologic	0.64 (0.58–0.70)	1.02 (0.95–1.09)
Gastrointestinal	1.18 (1.02–1.36)	1.59 (1.46–1.73)
Malignancy	0.85 (0.75–0.97)	1.18 (1.10–1.26)
Metabolic and renal	1.25 (1.10–1.43)	1.58 (1.38–1.79)
Other	0.93 (0.84–1.03)	0.99 (0.91–1.08)
Coma on admission	0.87 (0.76–0.99)	
Hospital length of stay		
1–15 days	Reference	Reference
16–30 days	1.44 (1.34–1.54)	1.21 (1.14–1.27)
31–45 days	1.62 (1.47–1.78)	1.43 (1.34–1.53)
> 45 days	1.58 (1.43–1.75)	1.56 (1.46–1.68)
Discharge destination		
Home	Reference	Reference
Other hospital	0.48 (1.47–1.78)	0.53 (0.50–0.57)
Skilled nursing facility	1.46 (1.26–1.69)	1.46 (1.23–1.73)
Others	1.38 (1.02–1.65)	0.88 (0.70–1.08)
Stress ulcer prophylaxis	–	1.22 (1.15–1.29)
pRBC infusion	1.16 (1.05–1.28)	1.43 (1.37–1.51)
Low-dose steroid	1.14 (1.01–1.29)	1.08 (1.00–1.16)
Anticoagulation	0.81 (0.76–0.87)	–
TPN	–	1.05 (1.00–1.09)
Antibiotics	1.09 (1.02–1.17)	–
RRT	1.28 (1.15–1.41)	1.24 (1.15–1.34)
Ventilation	1.18 (1.09–1.27)	–
Tracheostomy	–	0.83 (0.74–0.95)

*ICU* intensive care unit, *OR* odds ratio, *CI* confidence interval, *CCI* Charlson comorbidity index, *pRBC* packed red blood cells, *FFP* fresh frozen plasma, *TPN* total parental nutrition, *RRT* renal replacement therapy

## Discussion

In a large database of acute care hospitals in Japan, the key findings show that the overall cumulative 30-day unplanned hospital readmission rate following ICU admission was 3.4% (medical cases, 3.8%; surgical cases, 3.3%). For both medical and surgical patients, CCI; category of primary diagnosis (respiratory, gastrointestinal, and metabolic and renal); hospital LOS; discharge destination (skilled nursing facility); and receipt of pRBC transfusion, low-dose steroids, or RRT were associated with higher rates of unplanned hospital readmission.

Hospital readmission has become a major concern, especially in the realm of critical care medicine, as hospital readmissions affect patient quality of life, morbidity, and mortality [2, 4, 11, 12]. In addition, Krumholz and others [1, 13] proposed the idea of a post-hospital syndrome, namely, that patients become vulnerable to new health impairments for a transient period following hospitalization as a result of physical and cognitive disability, nutritional impairment, sleep deprivation, and continued delirium that increases their susceptibility to further illness post-discharge. While the spectrum of readmission diagnoses is largely diverse, respiratory infection, heart failure, digestive disorders, and renal disorders are common reasons for readmission [1, 14]. In fact, our findings showed that the proportions of respiratory, gastrointestinal, and metabolic and renal reasons for ICU admission were higher for readmission than for initial admission. Critically ill survivors of recent hospitalization are more vulnerable [15] and have a higher risk of unplanned hospital readmission [2]. Therefore, appropriate methods to identify patients at higher risk of readmission are essential to improve quality of care after discharge.

We found that the cumulative prevalence proportion of 30-day unplanned hospital readmission (3.6%) was similar to the reported proportion of heart failure in the DPC database [16]. On the other hand, a recent study using an administrative database in New York State revealed that the cumulative incidence of early unplanned rehospitalization within 30 days of discharge for survivors of critical illness was 16.2% [2]. Among all acute care, nonfederal hospitals in California, the all-cause 30-day readmission rates were 20.4, 23.6, and 17.7% for sepsis, congestive heart failure, and acute myocardial infarction, respectively [17]. Our results suggest that there is a discrepancy between re-hospitalization rates in Japan and the USA. Several possible factors, such as the Japanese social context, the universal health care system, and the demographics of the cohort, may explain why critically ill survivors in Japan showed lower rehospitalization rates than those in the USA. Notably, hospital LOS would be an important reason. Patients in Japan are generally hospitalized for longer periods than patients in the USA [18]; the LOS in Japan was twice as long as in the USA in one report [19]. In Japan, general wards are often used to provide rehabilitation and nursing care in addition to acute medical care for prolonged periods [16]. This practice might mitigate post-hospital syndrome and reduce gaps in outpatient care. Furthermore, we included patients under 60 years old; this group has the lowest rate of hospital readmission [2]. These factors contributed to the lower unplanned hospital readmission rates observed in Japan in the present study.

To examine the difference between medical and surgical cases, we divided ICU survivors into two groups; the rate of hospital readmission for surgical cases was lower than for medical cases. On the other hand, Hua et al. showed that non-surgical and surgical ICU survivors had the same cumulative rehospitalization rate (16.5% for non-surgical vs 15.9% for surgical; *P* = .87) [2]. Unlike hospital readmission of medical patients, readmission of surgical patients often results from delayed recognition of surgical procedure complications [20, 21]. Thus, the shorter the hospital LOS in general, the greater the possibility that complications will be identified after discharge. However, early medical follow-up of patients who are discharged after a surgical procedure is often performed in Japan [22], leading to lower rates of unplanned hospital readmission [5].

We identified several predictors associated with increased 30-day unplanned hospital readmission. Longer hospital LOS and discharge to skilled nursing facilities were identified as predictors in other studies assessing early rehospitalization [2, 5, 23–25]. Longer hospital LOS during the initial hospitalization is indicative of a more critically ill patient or the development of postoperative complications that might lead to rehospitalization [2, 5, 24, 26]. Additionally, past research has suggested that hospital readmission of patients discharged to skilled nursing facilities is higher than that of patients discharged to other destinations [23, 27]. A significant variation exists in the quality of care across skilled nursing facilities in Japan [28] and there is a paucity of reported data about care metrics. Better inpatient care, more complete predischarge resolution of certain problems, more effective care transitions to the home/community setting, and closer follow-up after discharge are important ways to reduce hospital readmissions.

Measures to prevent re-hospitalization remain elusive, as Walraven et al. in a recent systematic review reported that only 27% of hospital readmissions were preventable [29]. While most of predictors for hospital readmission in the present study can be considered as markers of severity of illness, RBC transfusion, which is possibly associated with increased risk of nosocomial infection, acute lung injury and acute kidney injury, [30] will be a potential target for intervention to reduce readmission rate. Future studies will be required to confirm this hypothesis.

### Strengths and limitations

There are several strengths to our study. First, we were able to investigate 30-day unplanned hospital readmissions in a large nationwide cohort of ICU survivors in Japan. As planned hospital readmissions do not reflect poor-quality care [5, 31, 32], we analyzed only unplanned hospital readmission as the primary outcome in this study. Second, adult patients of all ages were available in the DPC database, which accounted for whole adult ICU population. Third, the outcomes of and reasons for patients being readmitted to hospital were evaluated. This allowed us to identify the timing of hospital readmission and to evaluate the mortality associated with these hospital readmissions in the 30 days after discharge following the index hospitalization.

The present study also has several limitations. First, as our data source was an administrative database, we do not have information regarding patients who were readmitted to other hospitals, or information regarding out-of-hospital deaths. Also, we were not able to link data among patients transferred to another hospital. Hence, the true readmission rate may have been underestimated. However, death within 30 days post-discharge is a rare event [31]. Second, because our data were from Japan, our findings may not be generalizable to other countries with different healthcare systems, although some predictors such as longer index hospitalization, and discharge to a skilled nursing in the present study were also identified in other studies in other countries [2, 33, 34]. Third, data on some of the potential predictors, such as laboratory data, functional status, degree of social support, and adherence to medical treatment, were unavailable. Furthermore, our risk adjustment may have been limited due to the lack of general severity scoring models (Acute Physiology and Chronic Health Evaluation, Simplified Acute Physiology Score, or Sequential Organ Failure Assessment score), although we used treatments and procedures during the ICU stay as the surrogate measure. Fourth, information of end-of-life decisions to limit life-sustaining therapies was not available in our data, which could affect clinicians’ decisions not to readmit those patients. Fifth, information with regards to ICU and hospital discharge policy (senior decision or not, criteria, contribution of social workers, or relation with the family members) was unavailable. Finally, data on quality of care by medical professionals in the ICU that contributed to the care of each patient were lacking.

## Conclusion

In a large national database in Japan, the prevalence proportion of unplanned rehospitalization within 30 days of hospital discharge following ICU admission was 3.4%. We also found that critically ill patients with a higher CCI score; whose initial category of primary diagnosis was respiratory, gastrointestinal, or metabolic and renal; who had a longer hospital LOS; who were discharged to a skilled nursing facility; or who required pRBC infusion, low-dose steroids, or RRT during the index admission had a higher risk of 30-day unplanned hospital readmission. Further studies are required to improve readmission prediction modeling and targeted interventions for high-risk patients.

## Additional files

Additional file 1: **Table S1.** International classification the codes used to categorize the primary diagnosis. **Table S2.** Risk factors associated with 30–day unplanned hospital readmission of intensive care unit surgical patients for planned initial admission and urgent initial admission. (DOCX 25 kb)

Additional file 2: **Figure S1.** Distribution of the timing of 30-day readmission of medical or surgical intensive care unit survivors. The denominator was 5583 for medical intensive care unit survivors and 11,142 for surgical intensive care unit survivors. (PPTX 40 kb)

Additional file 3: **Figure S2.** Receiver operating characteristic curves for medical and surgical patients. (PPTX 48 kb)

## Abbreviations

CCI: Charlson comorbidity index
DPC: Diagnosis procedure combination
FFP: Fresh frozen plasma
ICD-10: International Classification of Diseases and Injuries 10th revision
ICU: Intensive care unit
IQR: Interquartile ranges
LOS: Length of stay
pRBC: Packed red blood cell
RRT: Renal replacement therapy
TPN: Total parental nutrition

## References

[1] Dharmarajan K, Hsieh AF, Lin Z (2013). Diagnoses and timing of 30-day readmissions after hospitalization for heart failure, acute myocardial infarction, or pneumonia. JAMA.

[2] Hua M, Gong MN, Brady J (2015). Early and late unplanned rehospitalizations for survivors of critical illness*. Crit Care Med.

[3] Hofhuis JG, Spronk PE, van Stel HF (2008). The impact of critical illness on perceived health-related quality of life during ICU treatment, hospital stay, and after hospital discharge: a long-term follow-up study. Chest.

[4] Wunsch H, Guerra C, Barnato AE (2010). Three-year outcomes for Medicare beneficiaries who survive intensive care. JAMA.

[5] Jencks SF, Williams MV, Coleman EA (2009). Rehospitalizations among patients in the Medicare fee-for-service program. N Engl J Med.

[6] Sacks GD, Dawes AJ, Russell MM (2014). Evaluation of hospital readmissions in surgical patients: do administrative data tell the real story?. JAMA Surg.

[7] Umegaki T, Nishimura M, Tajimi K (2013). An in-hospital mortality equation for mechanically ventilated patients in intensive care units. J Anesth.

[8] Ohnuma T, Shinjo D, Fushimi K (2016). Hospital mortality of patients aged 80 and older after surgical repair for type a acute aortic dissection in Japan. Medicine (Baltimore).

[9] Sundararajan V, Quan H, Halfon P (2007). Cross-national comparative performance of three versions of the ICD-10 Charlson index. Med Care.

[10] Shigematsu K, Nakano H, Watanabe Y. The eye response test alone is sufficient to predict stroke outcome—reintroduction of Japan coma scale: a cohort study. BMJ Open. 2013;3 10.1136/bmjopen-2013-002736 PMC3641437 23633419

[11] Lilly CM, Zuckerman IH, Badawi O (2011). Benchmark data from more than 240,000 adults that reflect the current practice of critical care in the United States. Chest.

[12] Garland A, Olafson K, Ramsey CD (2013). Epidemiology of critically ill patients in intensive care units: a population-based observational study. Crit Care.

[13] Krumholz HM (2013). Post-hospital syndrome—an acquired, transient condition of generalized risk. N Engl J Med.

[14] Vashi AA, Fox JP, Carr BG (2013). Use of hospital-based acute care among patients recently discharged from the hospital. JAMA.

[15] Harvey MA (2012). The truth about consequences—post-intensive care syndrome in intensive care unit survivors and their families. Crit Care Med.

[16] Aizawa H, Imai S, Fushimi K (2015). Factors associated with 30-day readmission of patients with heart failure from a Japanese administrative database. BMC Cardiovasc Disord.

[17] Chang DW, Tseng CH, Shapiro MF (2015). Rehospitalizations following sepsis: common and costly. Crit Care Med.

[18] Murthy S, Wunsch H (2012). Clinical review: international comparisons in critical care—essons learned. Crit Care.

[19] Sirio CA, Tajimi K, Taenaka N (2002). A cross-cultural comparison of critical care delivery: Japan and the United States. Chest.

[20] Graboyes EM, Liou TN, Kallogjeri D (2013). Risk factors for unplanned hospital readmission in otolaryngology patients. Otolaryngol Head Neck Surg.

[21] Shahian DM, He X, O'Brien SM (2014). Development of a clinical registry-based 30-day readmission measure for coronary artery bypass grafting surgery. Circulation.

[22] Honda M, Hiki N, Nunobe S, et al. Unplanned admission after gastrectomy as a consequence of fast-track surgery: a comparative risk analysis. Gastric Cancer. 2015; 10.1007/s10120-015-0553-5 26445945

[23] Mor V, Intrator O, Feng Z (2010). The revolving door of rehospitalization from skilled nursing facilities. Health Aff (Millwood).

[24] Kansagara D, Englander H, Salanitro A (2011). Risk prediction models for hospital readmission: a systematic review. JAMA.

[25] Sun A, Netzer G, Small DS (2016). Association between index hospitalization and hospital readmission in sepsis survivors. Crit Care Med.

[26] Donze J, Aujesky D, Williams D (2013). Potentially avoidable 30-day hospital readmissions in medical patients: derivation and validation of a prediction model. JAMA Intern Med.

[27] Neuman MD, Wirtalla C, Werner RM (2014). Association between skilled nursing facility quality indicators and hospital readmissions. JAMA.

[28] Nakanishi M, Hattori K, Nakashima T (2014). Health care and personal care needs among residents in nursing homes, group homes, and congregate housing in Japan: why does transition occur, and where can the frail elderly establish a permanent residence?. J Am Med Dir Assoc.

[29] van Walraven C, Bennett C, Jennings A (2011). Proportion of hospital readmissions deemed avoidable: a systematic review. CMAJ.

[30] Dupuis C, Sonneville R, Adrie C (2017). Impact of transfusion on patients with sepsis admitted in intensive care unit: a systematic review and meta-analysis. Ann Intensive Care.

[31] van Walraven C, Dhalla IA, Bell C (2010). Derivation and validation of an index to predict early death or unplanned readmission after discharge from hospital to the community. CMAJ.

[32] van Walraven C, Jennings A, Taljaard M (2011). Incidence of potentially avoidable urgent readmissions and their relation to all-cause urgent readmissions. CMAJ.

[33] Goodwin AJ, Rice DA, Simpson KN (2015). Frequency, cost, and risk factors of readmissions among severe sepsis survivors. Crit Care Med.

[34] Liu V, Lei X, Prescott HC (2014). Hospital readmission and healthcare utilization following sepsis in community settings. J Hosp Med.

