# Supplementary material for: Predictors associated with unplanned hospital readmission of medical and surgical intensive care unit survivors within 30 days of discharge
Source: J Intensive Care. 2018 Mar 1;6:14. doi: 10.1186/s40560-018-0284-x (PMC5831844; doi:10.1186/s40560-018-0284-x)
Supplement: Supplementary file 1 — Table S1. International classification the codes used to categorize the primary diagnosis. Table S2. Risk factors associated with 30–day unplanned hospital readmission of intensive care unit surgical patients for planned initial admission and urgent initial admission. (DOCX 25 kb) [file 40560_2018_284_MOESM1_ESM.docx]

**Supplemental Table 1 International classification the codes used to categorize the primary diagnosis**

| **Category** | **Constituent ICD–10 codes** |
| --- | --- |
| Cardiac | I00–I02, I05–I09, I10–I15, I20–I28, I30–I52, I95–I99 |
| Respiratory | J00–J06, J10–J18, J20–J22, J30–J39, J40–J47, J60–J70, J80–J86, J90–99 |
| Neurologic | I60–I69, I70–I79  G00–G09, G10–G13, G20–G26, G30–G32, G35–G37, G40–G47, G50–G59, G60–G64, G70–G73, G80–G83, G90–G99 |
| Gastrointestinal | K00–K14, K20–K31, K35–K38, K40–K46, K50–K52, K55–K63, K65–K67, K70–K77, K80–K87, K90–K93 |
| Malignancy | C00–C14, C15–C26, C30–C39, C40–C41, C43–C44, C45–C49, C50–C58, C60–C63, C64–C68, C69–C72, C73–C75, C76–C80, C81–C96, C97  D00–D09, D10–D36, D37–D48 |
| Metabolic and renal | E00–E07, E10–E14, E15–E16, E20–E35, E40–E46, E50–E64, E65–E68, E70–E90  N00–N08, N10–N16, N17–N19, N20–N23, N25–N29, N30–N39, N40–N51, N60–N64, N70–N77, N80–N98, N99 |

Abbreviations: ICD–10, International Classification of Diseases and Injuries, 10th revision.

**Supplemental Table 2 Risk factors associated with 30–day unplanned hospital readmission of intensive care unit surgical patients for planned initial admission and urgent initial admission**

|  | **Surgical**  **planned admission** | **Urgent admission** |
| --- | --- | --- |
|  |  |  |
|  | (n = 232, 597) | (n = 106, 593) |
| Variable | OR (95% CI) | OR (95% CI) |
| Teaching hospital | 0.82 (0.76–0.87) | 0.79 (0.70–0.89) |
| Hospital size: |  |  |
| <399 beds | Reference | – |
| 400–799 beds | 1.01 (0.94–1.08) | – |
| >800 beds | 0.90 (0.82–0.99) | – |
| CCI | 1.03 (1.02–1.05) | 1.08 (1.06–1.10) |
| Primary admission diagnosis category: |  |  |
| Cardiac | Reference | Reference |
| Respiratory | 1.55 (1.13–2.06) | 1.27 (1.07–1.51) |
| Neurologic | 1.16 (1.06–1.28) | 0.89 (0.80–0.99) |
| Gastrointestinal | 1.52 (1.28–1.80) | 1.30 (1.17–1.45) |
| Malignancy | 1.44 (1.32–1.58) | 1.09 (0.97–1.22) |
| Metabolic and renal | 2.08 (1.74–2.47) | 1.12 (0.91–1.35) |
| Other | 0.89 (0.77–1.01) | 0.97 (0.87–1.09) |
| Coma on admission | 2.85 (1.26–5.55) | – |
| Hospital length of stay: |  |  |
| 1–15 days | Reference | Reference |
| 16–30 days | 1.19 (1.11–1.27) | 1.13 (1.03–1.26) |
| 31–45 days | 1.32 (1.21–1.44) | 1.30 (1.17–1.46) |
| > 45 days | 1.54 (1.41–1.69) | 1.29 (1.16–1.44) |
| Discharge destination: |  |  |
| Home | Reference | Reference |
| Other hospital | 0.51 (0.45–0.57) | 0.51 (0.47–0.56) |
| Skilled nursing facility | 1.07 (0.63–1.69) | 1.35 (1.12–1.62) |
| Others | 0.77 (0.63–1.04) | 1.02 (0.74–1.37) |
| Stress ulcer prophylaxis | 1.25 (1.17–1.34) | 1.12 (1.02–1.23) |
| pRBC infusion | 1.44 (1.35–1.54) | 1.33 (1.23–1.43) |
| Anticoagulation | 1.08 (1.01–1.14) | – |
| TPN | 1.07 (1.02–1.14) | – |
| RRT | 1.27 (1.13–1.42) | 1.19 (1.07–1.33) |
| Tracheostomy | – | 0.82 (0.69–0.97) |

Abbreviations: ICU, intensive care unit; OR, odds ratio; CI, confidence interval; CCI, Charlson

comorbidity index; pRBC, packed red blood cells; FFP, fresh frozen plasma; TPN, total parental nutrition; RRT, renal replacement therapy.
