# Supplementary figures and images for: Predictors associated with unplanned hospital readmission of medical and surgical intensive care unit survivors within 30 days of discharge
Source: J Intensive Care. 2018 Mar 1;6:14. doi: 10.1186/s40560-018-0284-x (PMC5831844; doi:10.1186/s40560-018-0284-x)

## Slide 1
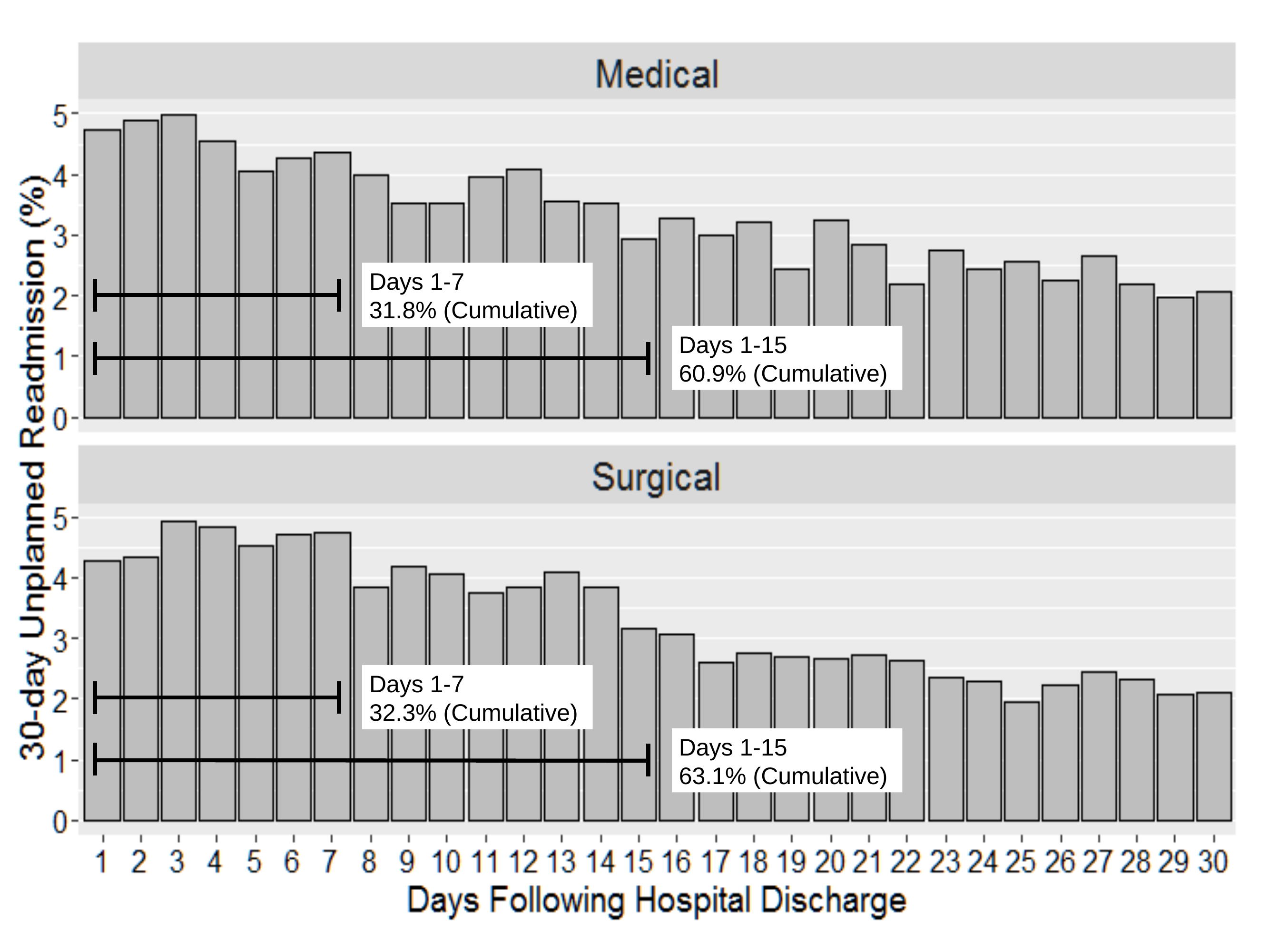

#
Days 1-7
31.8% (Cumulative)
Days 1-15
60.9% (Cumulative)
Days 1-7
32.3% (Cumulative)
Days 1-15
63.1% (Cumulative)

Supplement: Supplementary file 2 — Figure S1. Distribution of the timing of 30-day readmission of medical or surgical intensive care unit survivors. The denominator was 5583 for medical intensive care unit survivors and 11,142 for surgical intensive care unit survivors. (PPTX 40 kb) [file 40560_2018_284_MOESM2_ESM.pptx]

## Slide 1
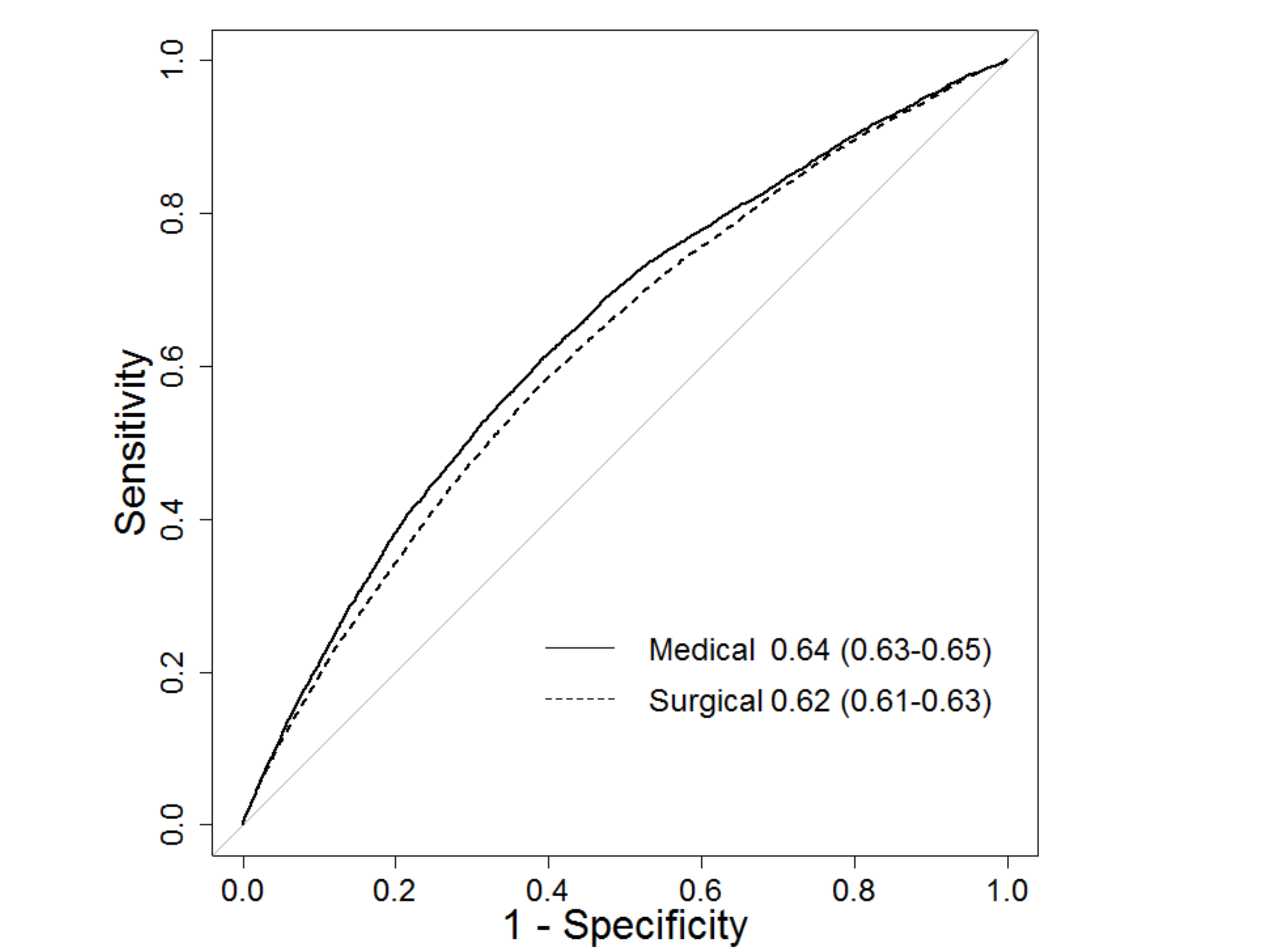

Supplement: Supplementary file 3 — Figure S2. Receiver operating characteristic curves for medical and surgical patients. (PPTX 48 kb) [file 40560_2018_284_MOESM3_ESM.pptx]
